# Short Telomeres Initiate Telomere Recombination in Primary and Tumor Cells

**DOI:** 10.1371/journal.pgen.1000357

**Published:** 2009-01-30

**Authors:** Tammy A. Morrish, Carol W. Greider

**Affiliations:** Department of Molecular Biology and Genetics, Johns Hopkins University School of Medicine, Baltimore, Maryland, United States of America; Brandeis University, United States of America

## Abstract

Human tumors that lack telomerase maintain telomeres by alternative lengthening mechanisms. Tumors can also form in telomerase-deficient mice; however, the genetic mechanism responsible for tumor growth without telomerase is unknown. In yeast, several different recombination pathways maintain telomeres in the absence of telomerase—some result in telomere maintenance with minimal effects on telomere length. To examine non-telomerase mechanisms for telomere maintenance in mammalian cells, we used primary cells and lymphomas from telomerase-deficient mice (mTR−/− and Eμmyc+mTR−/−) and CAST/EiJ mouse embryonic fibroblast cells. These cells were analyzed using pq-ratio analysis, telomere length distribution outliers, CO-FISH, Q-FISH, and multicolor FISH to detect subtelomeric recombination. Telomere length was maintained during long-term growth *in vivo* and *in vitro*. Long telomeres, characteristic of human ALT cells, were not observed in either late passage or mTR−/− tumor cells; instead, we observed only minimal changes in telomere length. Telomere length variation and subtelomeric recombination were frequent in cells with short telomeres, indicating that length maintenance is due to telomeric recombination. We also detected telomere length changes in primary mTR−/− cells that had short telomeres. Using mouse mTR+/− and human hTERT+/− primary cells with short telomeres, we found frequent length changes indicative of recombination. We conclude that telomere maintenance by non-telomerase mechanisms, including recombination, occurs in primary cells and is initiated by short telomeres, even in the presence of telomerase. Most intriguing, our data indicate that some non-telomerase telomere maintenance mechanisms occur without a significant increase in telomere length.

## Author Summary

Telomeres shorten with each cell division and are normally maintained by telomerase. Tumor cells typically up-regulate telomerase to maintain telomeres during rapid growth; however, some tumors lack telomerase and utilize alternative mechanisms to maintain the telomeres. Studies in yeast indicate that recombination contributes to telomere maintenance in the absence of telomerase. To examine whether recombination contributes to non-telomerase mechanisms for telomere maintenance in mouse and human cells, we utilized mouse primary and tumor cells, which were genetically deleted for telomerase. In addition, we assayed human primary cells that had short telomeres due to reduced telomerase activity. Our data indicate that cells with short telomeres have an increased level of telomere recombination, which can occur in telomeric or subtelomeric regions. We also found that, in addition to tumor cells, primary cells can utilize non-telomerase mechanisms for telomere maintenance. Lastly, we found that, unlike human telomerase negative tumors, some mechanisms of telomere maintenance do not result in dramatic telomere lengthening. Thus, mammalian cells likely utilize several different non-telomerase mechanisms for telomere maintenance. These findings will be useful for understanding the process of telomere lengthening in various types of human tumors.

## Introduction

Telomere length is maintained by the ribonucleoprotein complex telomerase [1]. However, telomerase expression in humans occurs primarily in early development, germ cells, and in stem cells and is not detected in primary cells [2],[3]. Most human tumor cells have detectable telomerase activity, however some proliferating tumors lack telomerase and thus maintain telomeres by alternative mechanisms that are collectively termed ALT for alternative lengthening of telomeres [4],[5]. While mTR−/− mice have a reduced frequency and rate of tumor formation, some tumors form and can grow rapidly in these mice [6],[7]. However, the mechanism by which tumors grow in the absence of telomerase is not known.

Telomerase deficient mice were initially generated by deleting the gene encoding the telomerase RNA (mTR) component [6]. Although mTR−/− mice lack telomerase activity, no phenotype is observed in the first generation, due to the long telomeres observed in laboratory mouse strains [6],[8]. When mTR−/− mice are bred, progressive telomere shortening occurs in successive generations. Early generation, mTR−/−G1, mice are obtained by crossing mTR+/− mice. Crossing the knockouts through successive generations results in mTR−/− G2–G6 generations. Late generation mTR−/− G4–G6 mice have short telomeres and show loss of fertility due to germ cell apoptosis. Wild-derived mouse strains such as CAST/EiJ have significantly shorter telomere length distributions, similar to humans [9]. CAST/EiJ mTR+/− mice bred for increasing generations show progressive telomere shortening and loss of tissue renewal capacity [10]. The phenotypes in the mTR+/− CAST/EiJ mice mimic the human genetic disease, dyskeratosis congenita, due to haploinsuffiency for telomerase [10]. Wildtype mice derived from an intercross between late generation heterozygous parents (termed WT*) have shorter telomeres and also display tissue renewal defects [10]. Thus, telomere shortening and consequent loss of tissue renewal capacity occurs in CAST/EiJ mice even in the presence of telomerase, and provides the opportunity to examine the effects of short telomeres in the presence of telomerase.

Several lines of evidence indicate that ALT occurs by DNA recombination in human tumors and immortalized cells [11]. First, the initial description of ALT demonstrated that the telomeres are exceptionally long and heterogeneous in human tumors and immortalized cell lines which lack telomerase [4]. Second, telomere lengths in ALT cells fluctuate during proliferation, and this fluctuation can be detected by examining the change in the telomere lengths at the p- and q-arm of the Y-chromosome in a rapidly growing culture [11]–[13]. Third, a unique plasmid sequence integrated as single copy at the telomere was found duplicated at other chromosomes following serial transfer of human ALT cells [14]. ALT associated nuclear promyelocytic leukemia bodies (APBs) are found in a subset of human ALT cell lines and contain various recombination proteins [15]. However it is uncertain what functional role APBs contribute to ALT mechanisms and they are often considered as only a marker for some ALT cell lines [16]. Finally, human ALT tumors show frequent telomere sister chromatid exchanges (T-SCEs), that are detectable using chromosome orientation FISH (CO-FISH) [17],[18].

Evidence that recombination contributes to telomere length maintenance was initially discovered in *Saccharomyces cerevisiae*. Yeast lacking an essential component of telomerase showed progressive telomere shortening and loss of viability, however survivors appeared after successive streaking of the colonies [19]. Studies of these yeast survivors showed that telomere recombination contributes to length maintenance, and requires the RAD52 pathway [19]. Survivors can be classified as Type I or Type II based on their telomere patterns and growth rate [20],[21]. Type I survivors require Rad51, Rad54, Rad55 and Rad57 [22]. The telomeres of Type I survivors are short and the cells have amplified Y′ sequence. They are likely generated by Rad51-dependent break-induced replication (BIR). Type II survivors grow much more rapidly than Type I survivors. They have elongated telomere sequence tracts, require Rad59, Rad50 and other components of the MRX complex, and are predominately generated by Rad51-independent BIR [22],[23]. Therefore, in yeast, telomere elongation in the absence of telomerase occurs mostly though BIR [22],[23]. Studies in *Kluyveromyces lactis* have also provided insight on telomere recombination mechanisms. In particular, *K.lactis* deleted for telomerase (*ter1*) showed that telomere recombination is initiated by short telomeres [24]. In mouse and human cells the T-SCE assay, which is frequently used to measure telomere recombination, will not detect recombination by BIR mechanisms. Further, T-SCEs are exchanges and thus will not result in net telomere elongation as occurs in BIR. Thus we sought to use other assays to examine telomere recombination in telomerase null mouse cells.

To examine the role of short telomeres during telomere recombination in mammalian cells we assayed cells using pq-ratios, outliers, CO-FISH, and Q-FISH from two different strains of telomerase deficient (mTR−/−) mice. We found that late passage CAST/EiJ mouse embryonic fibroblasts (MEFs) and Eμmyc+mTR−/− lymphomas with short telomeres, exhibit telomere maintenance with minimal changes to the overall length distribution. Consistent with telomere recombination, we observed an increase in pq-ratio changes and outliers in mouse cells with increasing numbers of short telomeres. We directly showed that subtelomeric recombination is increased in cells with elevated pq-ratio changes. These pq-ratio changes were seen associated with short telomeres even in telomerase positive cells, suggesting that telomerase itself does not protect against recombination. Our data suggest that, several distinct recombination-based mechanisms can contribute to telomere maintenance in mammalian cells.

## Materials and Methods

### Mice

C57BL/6J mTR−/− and CAST/EiJ mTR−/− were generated as described [6],[10]. Mice used for the intergenerational cross were generated as previously described [25],[26]. EμMyc+mTR−/− mice were bred and B-cell lymphomas were collected as previously described [7]. Mice were genotyped by Transnetyx (Cordova, TN). For serial transfers of tumors, B-cell lymphomas were isolated from mice and resuspended in PBS at 1×10^7^ cells/ml. A total of 1×10^6^ cells (0.1 ml) were subcutaneously injected into three SCID mice, two sites each (Taconic). All animals were housed and bred in a pathogen-free environment and procedures approved by the Institutional Animal Care and Use Committee at The Johns Hopkins University.

### Primary and Tumor Cell Culture and Metaphase Preparation

Splenocytes and bone marrow, from the tibias and femurs, were harvested from 8–10 week old animals. Bone marrow was collected by flushing the bones with 1× PBS, pH 7.4 (GIBCO) with a 23-gauge needle. Cells were resuspended in MarrowMax media (GIBCO), and immediately incubated with 0.1 µg/ml of KaryoMax Colcemid solution (GIBCO) for 20 minutes, and harvested for metaphase spread analysis. Cells were swelled in 75 mM KCl hypotonic solution at 37°C for 15 minutes, and fixed with (3∶1) methanol: acetic acid with three repeated exchanges prior to dropping onto slides. Splenocyte suspensions were generated using 70 µm Nylon cell strainers (BD Falcon), and were activated in RPMI 1640 with L-glutamine (GIBCO) supplemented with 1× penicillin-streptomycin-glutamine, 10% heat inactivated fetal bovine serum, 10 mM HEPES buffer, 1 mM sodium pyruvate (GIBCO), 1× non-essential amino acids (GIBCO), 50 µM ß-mercaptoethanol (Sigma), 10 µg/ml LPS (Sigma), 1 U/ml IL-2 (Roche), and 5 µg/ml ConA (Sigma). Splenocytes were cultured for 48 hours prior to the addition of 0.1 µg/ml of KaryoMax colcemid solution. Cells were incubated in colcemid for 20 minutes and metaphases prepared as described above. Lymphomas were collected from mice and single cell suspensions were generated using a 70 µm nylon cell strainer (BD Falcon). Cells were grown in a (1∶1) mixture of DMEM and Iscove's modified Eagle's media supplemented with 4 mM L-glutamine, 100 Units/ml of penicillin-streptomycin, 100 µM ß-mercaptoethanol, and 10% heat inactivated fetal bovine serum. Metaphases from human lymphocytes were acquired as previously described [27]. Mouse embryonic fibroblasts were harvested in 1× PBS containing 1× penicillin, streptomycin, and fungizone (PSF, Invitrogen) at embryonic day 13.5. Cells were incubated in 0.25% trypsin-EDTA for 20 minutes at 4°C, and further digested at 37°C for 5 minutes. Cells were transferred to DMEM containing 10% FCS and PSF. 24 hours post plating attached cells were washed in 1× PBS and new DMEM was added and split ∼24–48 hours later when cells were ∼80% confluent.

### Fluorescent *In Situ* Hybridization Including Quantitative (Q-FISH), CO-FISH, and Multicolor FISH

Metaphase spreads were processed for Q-FISH as previously described [26]. For CO-FISH, the cells were incubated for 24 hours in 30 µM of 5′-bromo-2′deoxyuridine (BrdU, Sigma) and 10 µM 5′bromo-2′deoxycytidine (BrdC, Berry & Associates). Bone marrow was incubated for 20 minutes and splenocytes for 2 hours in 0.2 µg/ml of colcemid and harvested for metaphases as described above. Metaphase spreads were rehydrated in 1× PBS pH 7.4 for 15 minutes and fixed in 4% formaldehyde in 1× PBS pH 7.4 for 2 minutes. All washes were done between treatments with 1× PBS and dehydrated in an ethanol series of 70%, 90% and absolute ethanol. Slides were treated with 1 mg/ml of pepsin at 37°C for 10 minutes, fixed in 4% formaldehyde in 1× PBS pH 7.4, dehydrated and treated with 500 µg/ml of RNase A in 2× SSC for 10 minutes at 37°C, and stained with 0.5 µg/ml of Hoechst 33258 (Sigma) for 15 minutes at room temperature, air-dried, and 100 µl of 2× SSC was added with a cover slip prior to a 30 minute UV exposure at 365 nm in an 1800 Stratalinker (Stratagene). Slides were digested with 3 U/µl of Exo III in 1× buffer (Promega) for 10 minutes at 37°C. Slides were hybridized with a telomere probe as described above for Q-FISH. Antibody staining to BrdU was used in order to avoid metaphases that underwent two rounds of replication. For BrdU staining, slides were washed in 2× SSC and incubated for 30 minutes at 37°C in a 1∶100 dilution of FITC anti-BrdU (Molecular Probes, Invitrogen) in PN buffer (0.1 M NaH2PO_4_/0.1 M Na_2_HPO_4_ pH 8.0, 1% Triton X-100) as described [28]. The slides were rinsed two times for 5 minutes each in PN buffer, prior to mounting with Vectashield. For multicolor FISH the subtelomeric clone (JHU1193) was acquired from Invitrogen (RPCI-23 391E5) and purified using HiPure Plasmid Filter Purification kit (Invitrogen). A mouse chromosome 2 specific paint probe was acquired from Applied Spectral Imaging (ASI). BAC DNA (1.5 ug) was labeled by Biotin-Nick Translation (Roche), ethanol precipitated with 15 µg mouse Cot-1 DNA (Invitrogen), and 1.5 µg of fish sperm DNA (Roche), and resuspended in 10 µl of formamide using a thermomixer at 37°C. 20 µl of probe hybridization buffer (20% Dextran sulfate, 2×SSC) was added, and the probe was denatured at 80°C for 5 minutes and pre-annealed at 37°C for one hour. Slides were pretreated as described for Q-FISH, denatured at 80°C in 70% formamide/2× SSC, and dehydrated in an ethanol series, prior to hybridization for 48 hour at 37°C. Slides are washed at 45°C in 50% formamide, 2× SSC for 3× 5 minutes and at 60°C in 0.1× SSC for 3× 5 minutes. Slides were dipped in 4× SSC/0.1% Tween 20 and 50 µl of denatured and pre-annealed chromosome 2 paint probe was hybridized for 48 hours at 37°C. Slides were washed in 0.4× SSC at 72°C for 3× 5 minutes and washed 2× 2 minutes each in 4× SSC/0.1% Tween 20. For detection of the BAC hybridization, slides were blocked in (3% BSA/4× SSC/0.1% Tween 20) for 30 minutes at 37°C for 30 minutes. Slides were washed for 2 minutes in 4× SSC/0.1% Tween 20 and streptavidin Alexa Fluor 488 (Molecular Probes, Invitrogen) conjugated antibody was diluted 1∶100 and incubated at 37°C for 45 minutes. Slides were washed in 4× SSC/0.1% Tween 20 at 45°C, 3× 5 minutes and dehydrated in an ethanol series. Slides were stained and visualized as described for Q-FISH.

### Pq-Ratios, Outliers, and Statistical Analysis

Telomere ratios were determined by initially measuring by Q-FISH the telomere lengths with TFL-Telo (Version 2.0) [29]. For each chromosome, the telomere signals were scored for location (p or q), and the final value of q/p was determined for each chromosome of multiple metaphases. 5-fold ratio values (q/p≥5 or q/p≤0.2) were plotted and normalized for the total number of chromosomes examined for each genotype. T-tests were used to determine statistical significance. Chromosomes with a single signal-free end were considered to have a q/p greater than 5-fold. The number of outliers was calculated by generating box plots using Stata 8.0. The box plots were generated using the Q-FISH values from the same metaphases examined for telomere ratio analysis. The test for statistical significance of outliers was done using the Wilcoxon rank sum test as described [30].

### Southern Analysis

Bone marrow and splenocytes were resuspended in 1% PBSa agarose at a final concentration of 1×10^7^ cells/ml, incubated in LDS (1% lithium dodecyl sulfate/100 mM EDTA pH 8.0, 10 mM Tris pH 8.) at 37°C O/N with constant agitation, and washed twice in 20% NDS for 2 hours at 37°C with constant agitation. Prior to digestion, plugs were washed twice in TE for 30 minutes and then washed twice in 400 µl of 1× Buffer #2 (NEB) for 30 minutes prior to MseI restriction digestion. Plugs were digested overnight and loaded on a 0.7% TAE agarose gel. Samples were run at 100V for 6–8 hours. Following denaturation (0.5 M NaOH/1.5 M NaCl) and neutralization (1.5 M NaCl/0.5 M Tris-HCL pH 7.4), the DNA was transferred in 20× SSC to a Nylon Membrane (Amersham Hybond N^+^) by weighting method overnight and cross-linked with UV Stratalinker (Stratagene). Pre-hybridization was done at 65°C in Church's buffer for 2 hours. A radioactive telomere probe was made by random-prime labeling using Prime-It II (Stratagene) with a slight modification. Briefly, 25 ng of a 500 bp telomeric 5′-TTAGGG/CCCTAA containing probe, acquired from EcoR1 digestion of JHU821 or 1 KB Plus DNA ladder (Invitrogen) was labeled using 33 µM of dATP, dTTP, 50 µCi of α-^32^P dCTP (3000 Ci/mmol) and 50 µCi α-^32^P dGTP (3000 Ci/mmol). Unincorporated nucleotides were removed using a G50 column (GE Healthcare). Labeled probe was counted and 10^6^ counts/ml (telomere probe) or 10^5^ counts/ml (ladder) was denatured at 100°C for 5 minutes and added to the pre-hybridization solution and hybridized overnight at 65°C. Membranes were washed 3× 15 minutes each in 6× SSC and 1%SDS at 65°C, and 3× 15 minutes each in 1× SSC and 1% SDS at 65°C and exposed to a phosphorimager screen and detected on a Fuji phosphorimager.

## Results

### Telomere Lengths Are Maintained with Minimal Changes to the Overall Length Distribution

**Figure 1:**
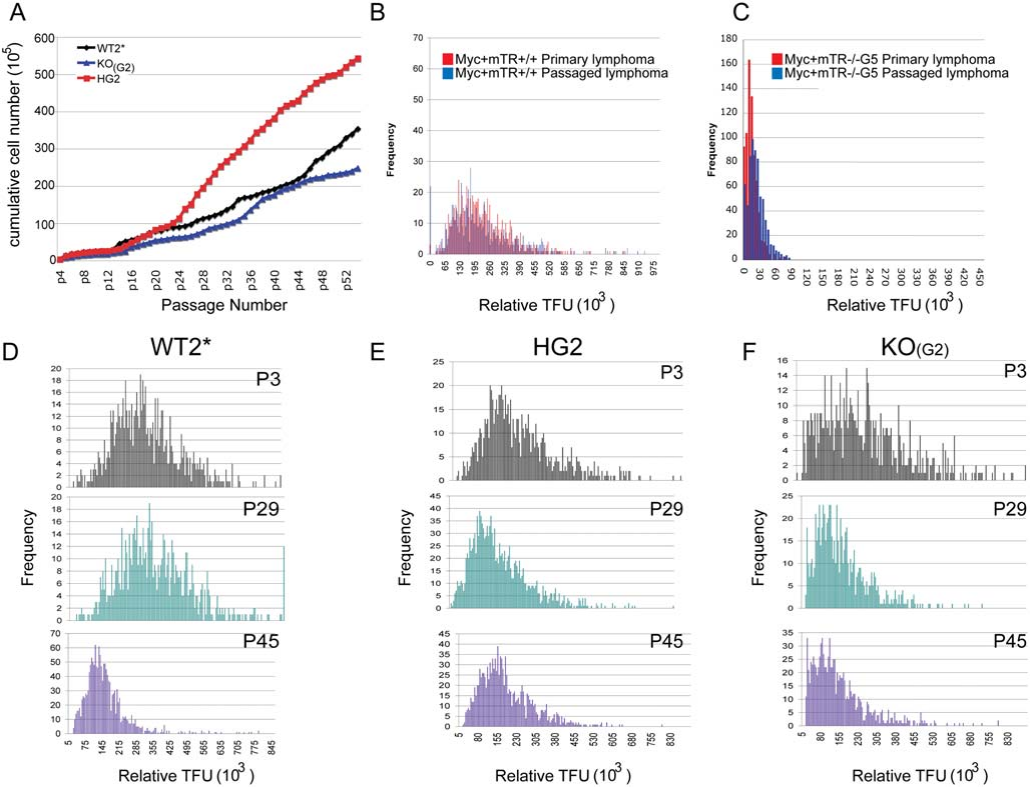
Telomere maintenance occurs in mTR−/− cells. A. Growth curves for CAST/EiJ MEFs derived from littermates from an HG1×HG1 cross. Cells were passaged according to previous reports [31]. Cell growth increased after a brief period of senescence and cells were considered immortalized after passage 16. B. Q-FISH on myc+mTR+/+ lymphomas serially passaged in SCID mice. C. Q-FISH on myc+mTR−/−G5 lymphomas serially passaged in SCID mice. D–F. Shown are the Q-FISH analysis of MEFs at passage 3 (p3), passage 29 (p29), and passage 45 (p45). The telomere lengths of both the HG2 and KO_(G2)_ were not dramatically lengthened. D. The WT2* mean telomere lengths are 3.1×10^5^, 3.5×10^5^, and 1.6×10^5^, the amount of signal free ends (SFEs) were, 1, 4, and 9, for p3, p29 and p45 respectively. E. For HG2 the mean telomere lengths were 2.6×10^5^, 2.0×10^5^, and 1.8×10^5^ and the SFEs were, 2, 23, and 19 for p3, p29 and p45 respectively. F. The mean telomere lengths for the KO_(G2)_ were 2.6×10^5^, 1.5×10^5^, and 1.3×10^5^, and the amount of SFEs were 16, 44, and 156 for P3, p29 and p45 respectively.

To examine the contribution of non-telomerase mechanisms to telomere maintenance, we serially passaged CAST/EiJ mTR−/− mouse embryonic fibroblasts (MEFs), which have short, homogeneous telomere lengths [9],[31]. After extensive passage, MEF cultures were immortalized as seen for mTR−/− MEFs on the C57BL/6J background (Figure 1A) [6]. Despite extensive passage of these MEFs, Q-FISH and Southern analysis indicated minimal changes to the telomere lengths in both mTR+/− and mTR−/− cell lines when compared to early passage cultures (Figure 1 D–F, compare p3 to p29 and p45, data not shown). These findings indicate that despite immortalization, MEFs lacking telomerase do not undergo extensive telomere lengthening. We reasoned that non-telomerase mechanisms for telomere maintenance may be occurring, similar to Type I survivors in yeast, which result in telomere maintenance without extensive lengthening.

We next asked whether such maintenance also occurs in transformed tumor cells lacking telomerase. We utilized Eμmyc+ transgenic mice that harbor the c-myc oncogene expressed by the B-cell specific Eμ promoter [32]. These mice invariably develop B-cell lymphoma and die from the tumor by six months of age. We generated Eμmyc+ mice with short telomeres. The tumor progression was dramatically reduced in the Eμmyc+ mTR−/− G6 mice with short telomeres [7]. However some tumors did form in these mice, and allowed for analysis using Q-FISH (Figure S1A). We first examined the telomere lengths in primary bone marrow and splenocytes from Eμmyc+mTR+/+, Eμmyc+mTR−/− G1, and Eμmyc+mTR−/− G4 and G6 tumor-free mice (Figure S1B and C). In primary cells, the telomere lengths were shorter in the late Eμmyc+mTR−/− generations compared to the early generations and when compared to Eμmyc+mTR+/+ cells. We next examined telomere lengths of the tumors that formed in Eμmyc+mTR+/+, Eμmyc+mTR−/− G1, and Eμmyc+mTR−/− G4 and G6 mice. We found that the telomerase negative tumors did not exhibit exceptionally longer telomeres when compared to primary bone marrow or splenocytes (Figure S1A–C). To examine if the telomeres could be maintained in the absence of telomerase, tumors with short telomeres (Eμmyc+mTR−/−G5) were serially passaged by transplantation into new recipient mice (Figure 1 B and C). Telomere lengths were examined, and again we observed minimal changes in telomere length in primary versus secondary lymphomas. Thus we conclude that both immortalized primary mouse cells and tumor cells can maintain telomere lengths in the absence of telomerase using non-telomerase mechanisms for telomere maintenance.

### Pq-Ratio Changes Are Increased in Primary and Tumor Cells with Short Telomeres

**Figure 2:**
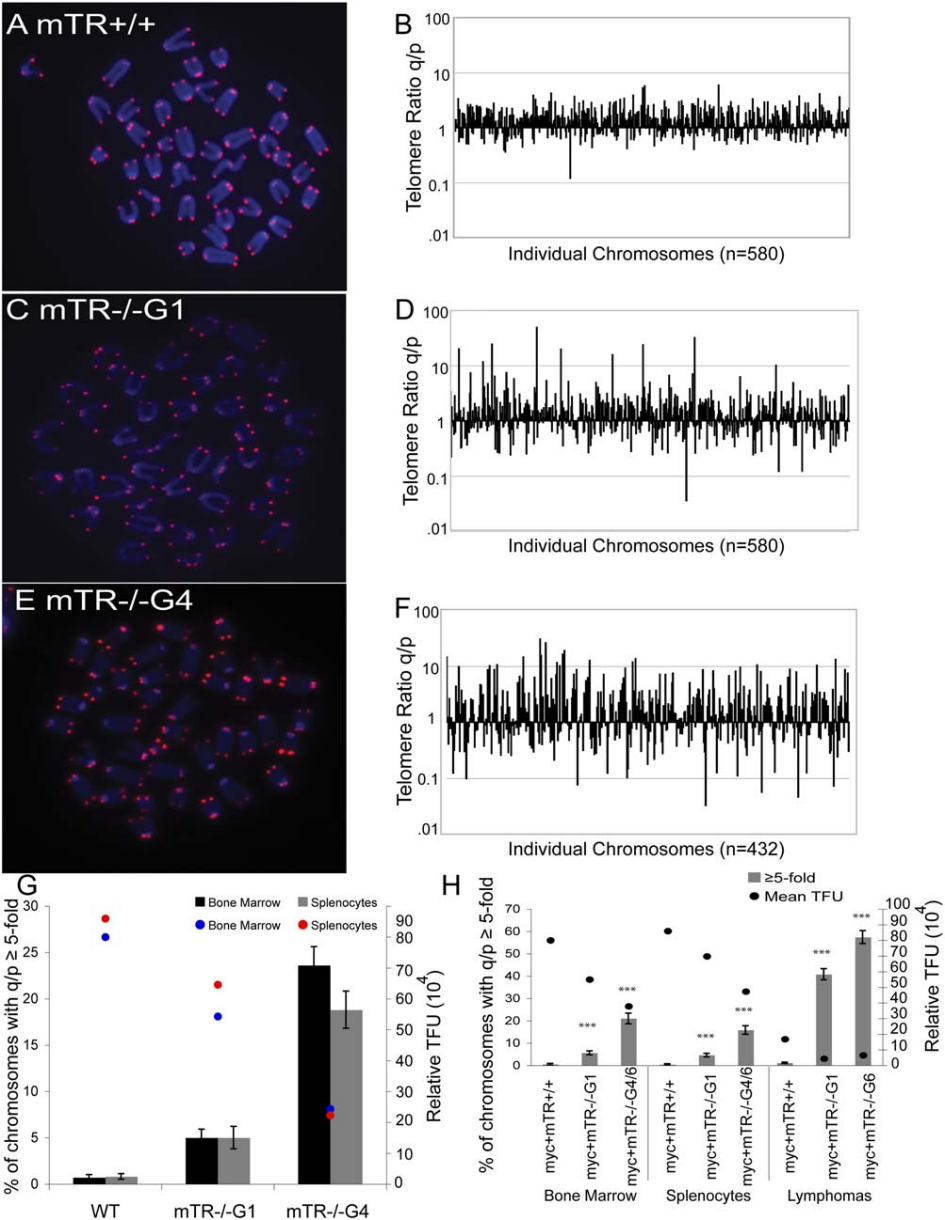
Pq-ratio changes are increased in early and late generation in mTR−/− cells with short telomeres. Telomere fluorescence *in situ* hybridization on metaphase spreads from primary bone marrow cells isolated from C57BL/6J mice, (A) mTR+/+, (C) mTR−/−G1 and (E) mTR−/−G4. Telomeres were hybridized with a Cy3-5′ (CCCTAA)_3_ telomere probe (red) and chromosomes are stained with DAPI (blue). Cy3-fluorescent intensity correlates with telomere length. B, D, F. The ratios (q/p) of the p and q telomere signals for each chromosome are plotted. 8–15 metaphases were examined for each genotype. Ratios (q/p) were calculated based on the telomere fluorescent intensity of the p- and q-arms of a given chromosome. The individual chromosomes assayed for each genotype are shown on the x-axis and the q/p ratio on the y-axis. The y-axis is on a log scale plot and excludes SFEs, since these values cannot be plotted on log scale. Changes in pq-ratios occurred for all metaphases, and were not specific to an individual metaphase, which is appreciated given that the chromosomes from each metaphase are graphed from left to right. G. The bar graph shows the percent of the total number of chromosomes with telomere ratio values of q/p≥5-fold (q/p≥5 or q/p≤0.2). Error bars represent the standard error of the mean. Values are shown for both primary bone marrow and splenocytes. Student T-tests, assuming α = .05, show statistical significance and were used to compare WT and mTR−/−G1 bone marrow (P = 3.8×10^−6^) and splenocytes (P = 8.3×10^−7^). Statistical significance was also observed between WT and mTR−/−G4 bone marrow (P = 3.6×10^−26^) and splenocytes (P = 8.8×10^−18^). Circles represent the mean telomere lengths plotted on the right y-axis. H. Pq-ratio changes determined for primary (pre-tumor) bone marrow and splenocytes as well as B-cell lymphomas plotted on the left y-axis. Error bars represent SEM. The circles represent the mean telomere length plotted on the right y-axis. Statistical significance was observed using a T-test (α = .05) between myc+mTR+/+ and myc+mTR−/−G1 primary bone marrow (P = 2.3×10^−8^) and splenocytes (P = 6.6×10^−7^). Similarly, statistical significance was observed between myc+mTR+/+ and myc+mTR−/−G4/G6 primary bone marrow (P = 1.4×10^−18^) and splenocytes (P = 3.3×10^−15^).

Since we observed that telomerase negative tumors and CAST/EiJ MEFs appear to be utilizing non-telomerase mechanisms to maintain telomeres, we sought to determine the basis of this telomere maintenance mechanism that occurred without significant changes to telomere lengths. We examined whether short telomeres may initiate the non-telomerase maintenance mechanisms operating in these cells. We utilized a previously described pq-ratio assay, which can account for different types of telomere recombination mechanisms [12]. This assay is based on the Q-FISH assay, which quantitates the telomere length on metaphase spreads [29]. As cells divide in culture, both telomeres of a given chromosome should shorten by similar amounts. This results in a near constant telomere ratio at the p and q arms for each individual chromosome in a population of growing cells. However, if recombination occurs, it will alter the length of at least one telomere by a random amount. Thus the ratio of the telomere signal on the two ends of a chromosome that underwent recombination will differ from the other copies of that chromosome in the population. For pq-ratio analysis, we initially calculated the pq-ratio for mouse chromosome 1 in a population of growing cells (Figure S2A). Cells expressing telomerase had few changes in the telomere ratio (Figure S2B). Similar to human ALT cell lines, we observed that mouse tumor cells lacking telomerase were increased for changes in the pq-ratios [12] (Figure S2C). Given that this analysis of a single chromosome could be expanded to examine the changes within the entire set of chromosomes of a given metaphase, we proceeded by examining the pq-ratio of every chromosome in a minimum of ten different metaphases (Figure 2 and Figure S3). We first examined primary bone marrow from mTR+/+ mice and early and late generation mTR−/− mice (mTR−/−G1 and mTR−/−G4). Metaphase spreads were examined for telomere length using Q-FISH (Figure S1 D and E). Late generation mTR−/−G4 mice had significantly shorter telomeres than mTR+/+ or early generation mTR−/−G1 mice. We next examined the pq-ratios from metaphase spreads from a population of growing cells and plotted the ratio for each genotype (Figure 2A–F). To quantitate the changes in the pq-ratios, we focused on the percent of chromosomes that had a pq-ratio change of greater than 5-fold (Figure 2G). In mTR+/+ bone marrow the pq-ratios seldom changed from a value near 1 (Figure 2A, B, and G). Compared to mTR+/+, mTR−/−G1 primary bone marrow cells were significantly increased for changes in pq-ratios (Figure 2C, D and G). mTR−/− G4 cells with even shorter telomeres also showed a significant increase in the number of altered pq-ratios compared to mTR+/+ (Figure 2E, F and G). These changes in pq-ratios occurred in all metaphases examined, and were not specific to individual metaphases. Similar findings were also observed with splenocytes from the same genotypes (Figure 2G). The greater amount of changes in the pq-ratios in cells with shorter telomeres likely reflects non-gradual additions or deletions of telomere sequence that occurs during recombination.

We next asked whether Eμmyc+mTR−/− primary cells with short telomeres were also increased for changes in the pq-ratios. Comparisons between Eμmyc+mTR−/−G1 and Eμmyc+mTR+/+ primary bone marrow showed a 11-fold increase in variable pq-ratios (Figure 2H). We also observed a 40-fold increase in pq-ratio changes between Eμmyc+mTR−/− G4 and G6 compared to Eμmyc+mTR+/+ bone marrow (Figure 2H). Similar trends were observed in splenocytes (Figure 2H). Thus similar to mTR−/− primary cells with short telomeres, Eμmyc+ mTR−/− primary cells with short telomeres were increased for changes in the pq-ratios.

To determine whether similar changes in pq-ratios occurred in telomerase negative tumors, we examined Eμmyc+ tumors (Figure 2H and Figure S3). Eμmyc+mTR−/−G1 tumors showed a 37-fold increase in pq-ratios that changed compared to Eμmyc+mTR+/+ tumors. Eμmyc+mTR−/−G6 tumors showed a 52-fold increase compared to Eμmyc+mTR+/+ tumors. These findings suggest that telomerase deficient tumor cells with short telomeres are likely increased for telomere recombination. We then compared the number of pq-ratio changes in tumors and primary cells and found that tumors from both early Eμmyc+mTR−/− G1 and late Eμmyc+mTR−/− G4/G6 generation mice were increased in the amount of pq-ratio changes compared to the primary bone marrow from the same generation (Figure 2H). Eμmyc+ mTR+/+ tumors were also slightly increased for changes in the pq-ratios compared to Eμmyc+mTR+/+ primary cells, perhaps due to selection for increased recombination during growth of the tumor. Similar observations were made between the primary splenocytes and tumors. Our data indicate that telomerase negative tumor cells have an elevated amount of telomere recombination compared to primary cells. Furthermore, it illustrates that some non-telomerase telomere maintenance mechanisms have a minimal effect on the average telomere length.

### Outliers Are Increased in Primary and Tumor Cells with Short Telomeres

**Figure 3:**
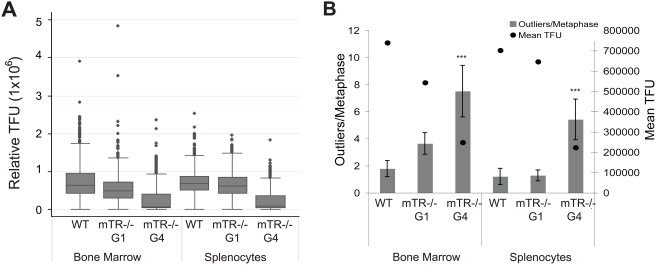
Late generation mTR−/− primary cells have a significantly greater number of telomere outliers. Data acquired from Q-FISH were used to generate box plots. The darkened line within the box represents the 50^th^ percentile, the median telomere length of the distribution. The box includes telomere length values and is approximately one standard deviation. The lines extending from outside the box represent approximately two standard deviations (95^th^ and 5^th^ percentile). Telomere lengths outside of these are outliers and appear as dots. Since some data points overlap, the total amount of outliers is graphically quantitated in B, and is normalized for the total number of metaphases examined. B. The graph shows the total number of outliers divided by the total number of metaphases (n) for each genotype plotted on the left y-axis. Error bars represent the standard error of the mean (SEM). Circles reflect the mean telomere length plotted on the right y-axis. Calculated p-values (α = .05) using the Wilcoxon rank sum test indicate a statistically significant difference between mTR+/+ and mTR−/−G4 bone marrow (P = .004). Similarly mTR−/−G4 splenocytes have a statistically greater number of outliers in comparison to mTR+/+ splenocytes (P = .008).

While the pq-ratio assay is very sensitive, the data may become biased as the telomeres shorten. Small changes on a short telomere may be over-represented and telomeres with no signal will not be represented at all. Thus we used a second statistical test to assay for length changes. We determined the distribution of the telomere lengths and quantitated the number of outliers (Figure 3A), which are telomere lengths more than two standard deviations from the median. These outliers numerically represent exceptionally long and short telomeres in the distribution.

The telomere length distribution acquired from Q-FISH for each genotype was determined and examined to identify outliers (shown as dots, Figure 3A). We found mTR−/−G4 bone marrow and splenocytes cells had a significantly greater number of outliers per metaphase compared to mTR+/+ cells (Figure 3B). This result is consistent with the findings observed with the pq-ratio analysis, and suggests that short telomeres may initiate telomere recombination.

### Eμmyc+mTR−/− Tumors Are Increased for Subtelomeric Recombination

**Figure 4:**
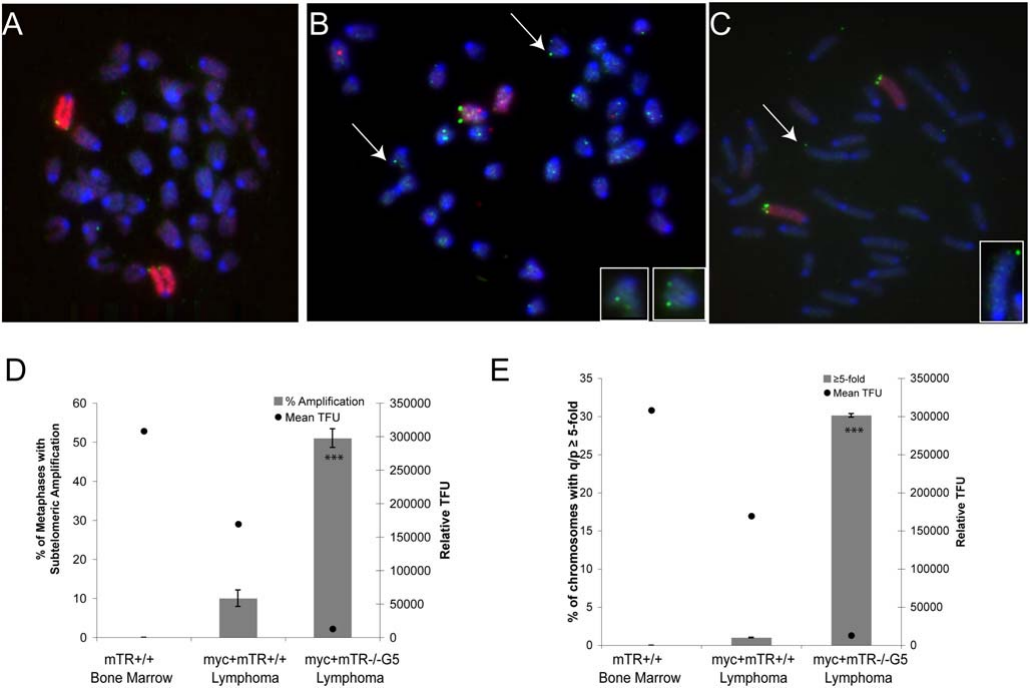
Subtelomeric recombination is frequent in Eμmyc+mTR−/−G5 tumors. Multicolor FISH using a BAC that maps to the subtelomeric region (green) of mouse chromosome 2 hybridized to metaphase spreads. Chromosome 2 was hybridized with a chromosome specific probe (red). Chromosomes with subtelomeric amplification are indicated with a white arrow. A. mTR+/+ bone marrow. B–C. Eμmyc+mTR−/−G5 tumors have amplified this subtelomeric sequence to other chromosomes, white arrows. D. Percent of metaphases with subtelomeric amplification (left, y-axis) and the mean telomere length (right, y-axis). Error bars represent the standard deviation. Subtelomeric amplification is increased in cells with short telomeres, and also appears at reduced levels in myc+mTR+/+ lymphomas. Fifty metaphases were scored for each genotype. Student T-tests, assuming α = .05, show statistical significance difference between the myc+mTR+/+ and the myc+mTR−/−G5 lymphomas (P = 5.0×10^−5^). E. Cells with an increased number of pq-ratio changes are also elevated for subtelomeric amplification. Error bars represent the standard deviation. Student T-tests, assuming α = .05, show statistical significance difference between the myc+mTR+/+ and the myc+mTR−/−G5 lymphomas (P = 6.8×10^−6^).

Our data suggest that abrupt changes in telomere length occur on short telomeres. To directly test whether recombination is occurring in these cells, we developed a subtelomeric recombination assay. We examined the mouse genome sequence for unique loci contained in subtelomeric regions, and identified a terminal BAC clone located at H4 on mouse chromosome 2, which also contained subtelomeric sequences directly adjacent to telomere repeats in the genome. We initially hybridized this sequence to mTR+/+ cells to confirm the copy number of this subtelomeric BAC clone and found the clone hybridized to only two chromosomal termini in wildtype bone marrow metaphase spreads (Figure 4A). We reasoned that if recombination in the telomeric region occurs in the subtelomeric regions, as it does in yeast, amplification and transfer of this sequence to the telomeres of other chromosomes would occur. To determine the frequency of this sequence amplification and transfer, we hybridized metaphase spreads with both the BAC clone and a chromosome 2 specific paint probe (Figure 4B and C). We first examined the Eμmyc+mTR−/− G5 lymphomas. Consistent with subtelomeric recombination, we observed 51% of the metaphases had amplified this subtelomeric region in the Eμmyc+mTR−/− G5 lymphomas with short telomeres (Figure 4D). We observed no metaphases with amplified subtelomeric sequence in primary mTR+/+ bone marrow. In the Eμmyc+mTR+/+ lymphomas there was a low level of amplification of this sequence likely due to increased recombination in the tumors (10%). In addition we observe the amount of subtelomeric recombination in mTR−/− MEFs, when restored for mTR have a reduced number of changes in both pq-ratios and the frequency of subtelomeric recombination (unpublished data). Given that early passage primary cells lack fusions, its unlikely that bridge fusion breakage cycles contribute to the amplification of the sequence. This subtelomeric amplification and transfer of a unique locus to additional chromosomes correlated directly with increased changes in the pq-ratios (Figure 4E), suggesting that BIR could account for both processes.

### Short Telomeres in mTR−/− Primary Cells from an Intergenerational Cross Show Increased Recombination

To more specifically examine whether short telomeres are substrates for recombination, we used mice from an intergenerational cross [25],[26]. All progeny from this type of cross inherit chromosomes with 50% short and 50% long telomeres. Previous studies showed that when late generation mTR−/− mice were crossed with mTR+/− mice, the shortest telomeres were specifically elongated in mTR+/− mice [25].

**Figure 5:**
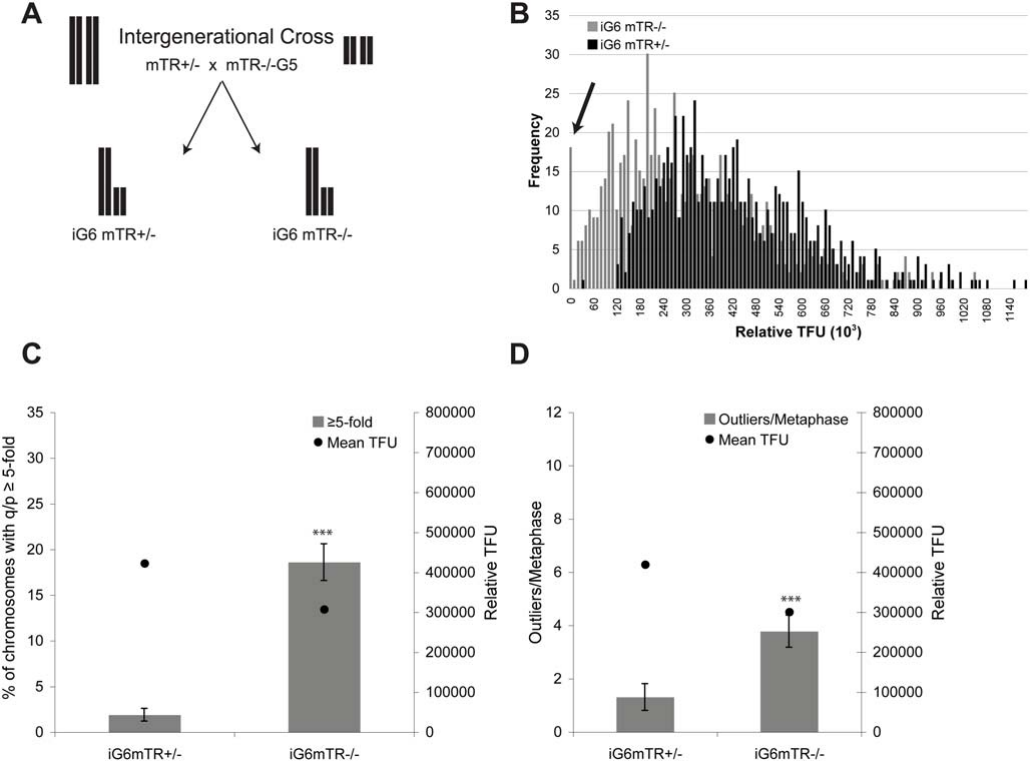
iG6 mTR−/− cells from an intergenerational cross have an increase in pq-ratio changes and outliers. A. The breeding scheme used to generate intergenerational (iG6) mice results in progeny with half short and half long telomeres. B. Q-FISH on primary bone marrow cells isolated from mTR−/− iG6 and mTR+/−iG6 mice. The arrow points to the class of shortest telomeres that persist in the iG6 mTR−/− mice. C. Summary of pq-ratio analysis on bone marrow cells. Circles represent the mean telomere length. Using a T-test (α = .05) statistical significance was observed between the number of ratios with q/p≥5-fold for mTR−/− iG6 and mTR+/− iG6 bone marrow cells (P = 2.2×10^−15^). D. Box plot analysis used to determine the number of telomere “outliers” is plotted on the left y-axis. Circles represent the mean telomere length and are plotted on the right y-axis. Using a Wilcoxon rank sum test (α = .05), mTR−/− iG6 bone marrow cells have a statistically greater number of outliers compared to mTR+/− iG6 (P = .003).

For our analysis, we crossed late generation telomerase mTR−/− G5 mice with mTR+/− mice (Figure 5A). Bone marrow was harvested from mTR−/− iG6 and mTR+/− iG6 mice and analyzed by Q-FISH. We observed that the shortest telomeres from mTR+/− iG6 mice were extended, while critically short telomeres persisted in mTR−/− iG6 mice (Figure 5B, arrow). When we examined the pq-ratios, we found a significant increase in the pq-ratios that changed in mTR−/− iG6 mice compared to mTR+/− iG6 mice (Figure 5C). Outlier analysis of telomere lengths from this intergenerational cross also indicated a significantly greater number of outliers per metaphase in the mTR−/− iG6 mice compared to the mTR+/− iG6 mice (Figure 5D). These data strongly suggest that short telomeres are substrates for recombination. We also noted that the mTR+/− iG6 mice displayed a small amount of pq-ratio changes and had some outliers. This small, yet detectable amount of pq-ratio changes and outliers in the mTR+/− intergenerational mice suggested that perhaps telomere recombination occurs in the presence of telomerase.

### Short Telomeres Initiate Recombination in CAST/EiJ Mice, Even in the Presence of Telomerase

**Figure 6:**
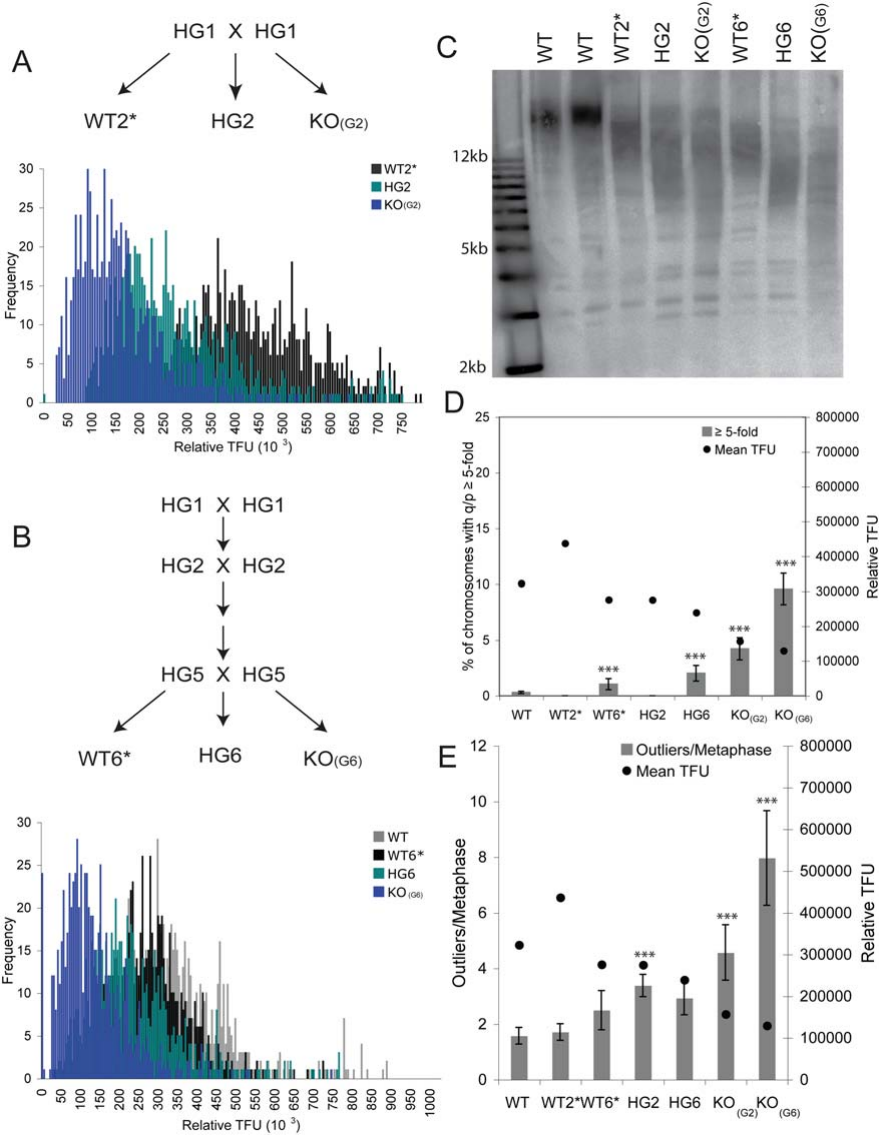
Short CAST/EiJ telomeres even in the presence of telomerase show increased pq-ratio changes. A and B. Q-FISH on primary bone marrow isolated from early generation (HG1×HG1) and late generation (HG5×HG5) CAST/EiJ mice. The breeding scheme used to generate the CAST/EiJ mice is shown above the Q-FISH plots. C. Southern analysis on bone marrow cells isolated from the same mice shown in A and B. D. The pq-ratios of primary bone marrow cells are plotted as described above. The percent of the total number of chromosomes with telomere ratio values q/p≥5-fold is plotted on the left y-axis. The circles represent the mean telomere lengths of each genotype plotted on the right y-axis. A T-test (α = .05) was used to determine statistical significance between WT bone marrow cells versus WT6* (P = .04), HG6 (P = .002), KO_(G2)_ (P = 8.8×10^−5^), and KO_(G6)_ (P = 5.9×10^−11^). E. Summary of outliers generated from box plots. The outliers per metaphase are plotted on the left y-axis. The circles represent the mean telomere length plotted on the right y-axis. A Wilcoxon rank sum test (α = .05) was used to calculate statistical significance between WT versus HG2 (P = .009), KO_(G2)_ (P = .016) and KO_(G6)_ (P = .0001).

To test more directly whether some short telomeres may recombine in the presence of telomerase, we utilized mTR+/− CAST/EiJ mice. As described previously, telomere shortening occurs in late generation mTR+/+ and mTR+/− (termed WT#* and HG#) CAST/EiJ mice [10]. We assayed littermates from both early and late generation heterozygous intercrosses, including a cross from the first generation of heterozygous mice (HG1) which yielded WT2*, HG2, and knockout KO_(G2)_ mice and a cross of HG5 parents which yielded WT6*, HG6, and KO_(G6)_ mice (Figure 6A and B). Since telomeres are shorter and more homogenous in the CAST/EiJ strain compared to C57BL/6J, we examined telomere length by both Southern blotting and Q-FISH (Figure 6C and Figure S4 A–C) [9]. We observed that the WT* mice from the late generation HG5 intercross (WT6*), had shorter telomeres than true wildtype (WT) and early generation WT2* mice. We also observed that the telomeres from the late generation HG6 mice were shorter than HG2 mice. We next examined the pq-ratio changes and the number of telomere outliers. WT6*, HG6, and both KO_(G2)_ and KO_(G6)_ mice, had a significant increase in changed pq-ratios compared to WT mice (Figure 6D and Figure S4D). These findings illustrate pq-ratios changes increase in cells with short telomeres, even in the presence of telomerase. Consistent with this finding, we also observed an increase in the amount of outliers in bone marrow and splenocytes cells with short telomeres (Figure 6E and Figure S4E). Together the pq-ratio and outlier analysis suggest that short telomeres may initiate recombination even when telomerase is present at wildtype levels as in the WT6*.

### Telomere Sister Chromatid Exchanges Are Detected in Only a Subset of Primary Cells with Short Telomeres

**Figure 7:**
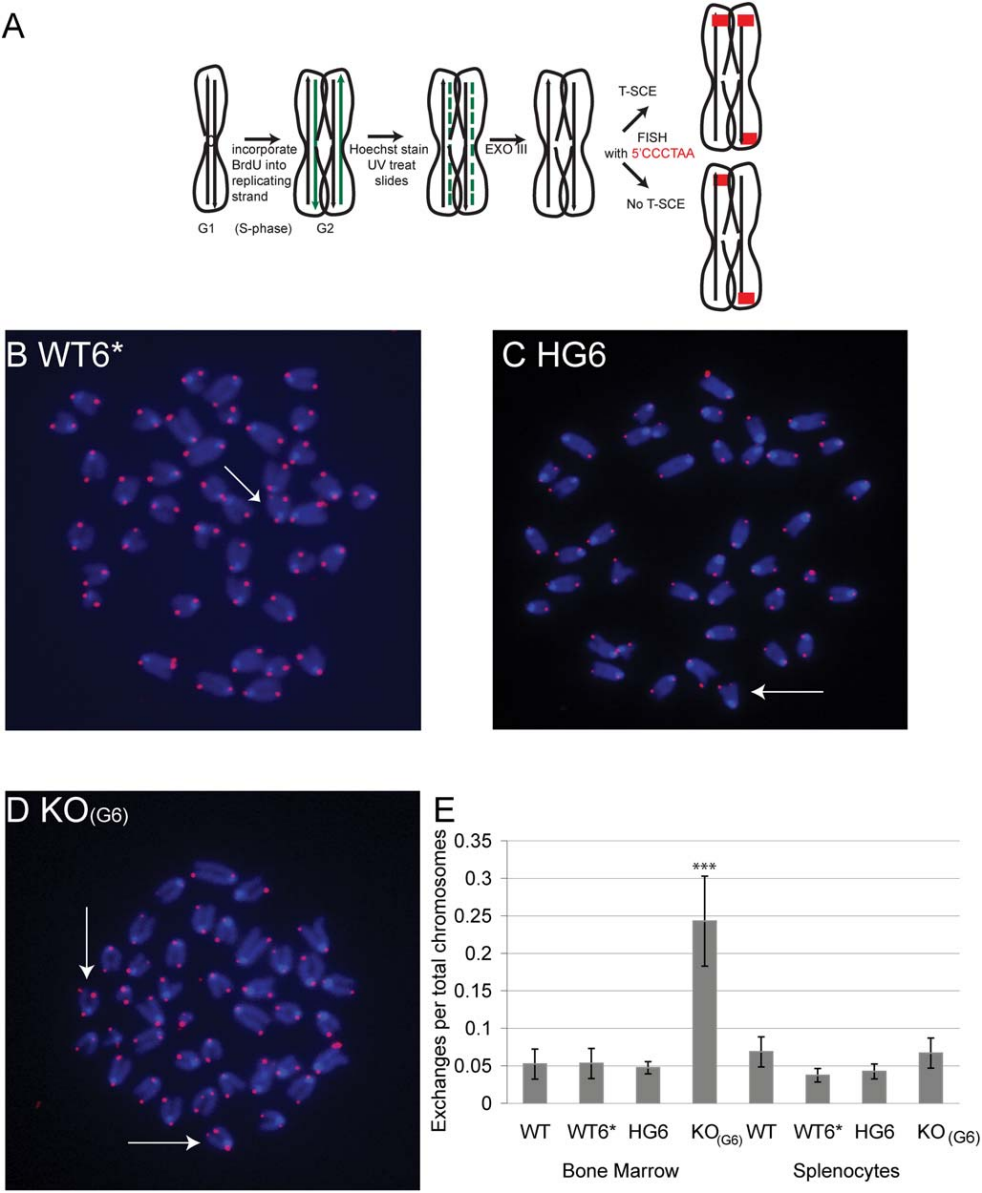
CO-FISH detects frequent exchanges in only some primary mTR−/− cells. A. A diagram of CO-FISH analysis. In the CO-FISH assay, cells are grown in the presence of 5-bromo-2-deoxyuridine (BrdU). After one round of replication one strand (green) has incorporated BrdU. Metaphases are treated with Hoechst and UV treated, which nicks the BrdU incorporated strand. The nicked strands are then degraded with an exonuclease, followed by hybridization with a telomere probe. B–D. CO-FISH on primary bone marrow cells isolated from the progeny of late generation (HG5×HG5) CAST/EiJ mice WT6* (n = 3), HG6 (n = 3) and KO_(G6)_ (n = 3). WT (n = 3) mice were also examined. E. Summary of T-SCEs in primary CAST/EiJ bone marrow cells. A T-test (α = .05) was used and showed a significant increase in the amount of T-SCEs in primary KO_(G6)_ bone marrow (P = .003) compared to WT bone marrow.

Since T-SCE has been documented in ALT cells, we wanted to determine whether this type of recombination could account for the pq-ratio changes occurring in primary cells. Using the CO-FISH assay we examined littermates from late generation (HG5×HG5) CAST/EiJ mice and WT CAST/EiJ mice for T-SCE. Primary bone marrow from WT, WT6*, HG6, and KO_(G6)_ mice was isolated and examined by CO-FISH (Figure 7). In WT6* and HG6 mice with short telomeres, we observed only a small amount of T-SCEs (0.05 T-SCEs/chromosome, Figure 7E). Similar frequencies were observed in bone marrow from wildtype mice. In the KO_(G6)_ bone marrow cells the amount of T-SCEs was significantly higher (0.25 T-SCEs/chromosome) when compared to WT6* and HG6. However, in splenocytes we observed a similar frequency of T-SCEs for all genotypes (0.04–0.07 T-SCEs/chromosome). The dissimilar amount of T-SCEs between cell types could be due differences in the type of recombination mechanism contributing to the telomere maintenance in splenocytes versus bone marrow. The difference in the number of T-SCEs in the WT6* and KO_(G6)_ bone marrow is unlikely due to differences in replication rate of these cells, since both genotypes have similar proliferation rates (Morrish, Armanios and Alder, unpublished data). Instead the increase in T-SCEs in KO_(G6)_ bone marrow cells compared to WT6* implies that T-SCEs may be one type of recombination mechanism that occurs with short telomeres.

### Short Telomeres in Human Cells Show Evidence of Increased Recombination

**Figure 8:**
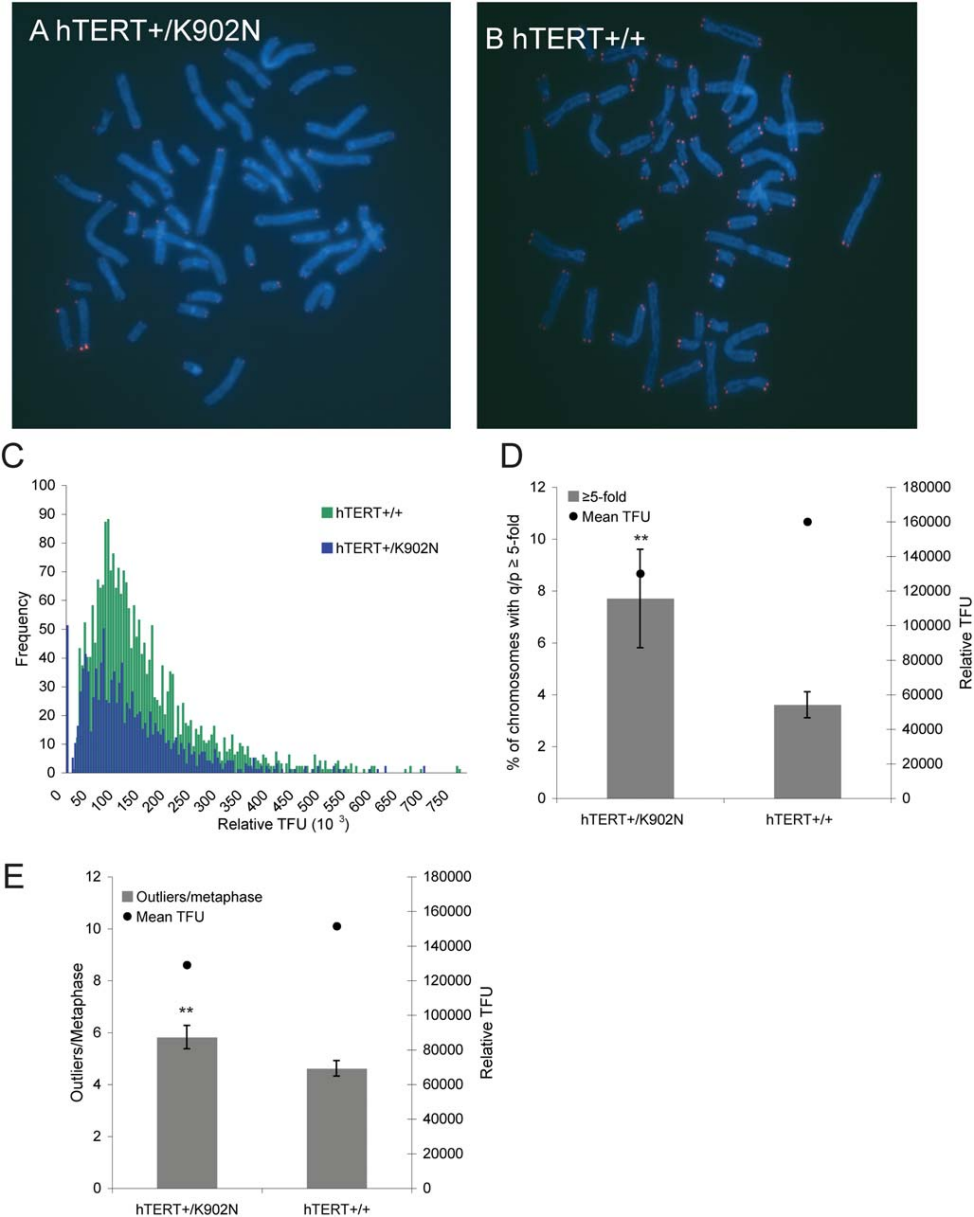
Lymphocytes from hTERT+/− individuals are increased for pq-ratio changes and outliers. A and B. Shown are the Q-FISH results on lymphocytes from various individuals in a family harboring a mutation in hTERT (hTERT+/K902N) or from non-carriers hTERT+/+. C. Summary of Q-FISH analysis from related individuals with hTERT+/K902N (n = 4) and hTERT+/+ (n = 6) genotypes. D. The total number of chromosomes with telomere ratio values q/p≥5-fold is plotted on the left y-axis. The black circle denotes the mean telomere length plotted on the right y-axis. Using a T-test (α = .05), comparison of hTERT+/+ with hTERT+/K902N shows a significant difference (P = .02). E. The total number of outliers per metaphase is plotted on the left y-axis. Black circles represent the mean telomere length plotted on the right y-axis. Using a Wilcoxon rank sum test (α = .05) a significantly greater number of outliers were observed in hTERT+/K902N in comparison to hTERT+/+ (P = .034).

While human ALT tumor cells show greatly elongated telomeres, we wondered if primary human cells with short telomeres might utilize telomere recombination, without telomere lengthening. We thus examined both pq-ratios and outliers in lymphocyte cells from dyskeratosis congenita patients with short telomeres due to a mutation in hTERT (K902N) (Figure 8A–C) [27]. Individuals with short telomeres had a significantly greater amount of changes in pq-ratios in comparison to non-carriers (hTERT+/+) in the family (Figure 8D). Analysis of the frequency of outliers per metaphase also demonstrated that carriers with short telomeres had a significantly greater number of outliers compared to non-carriers with longer telomeres (Figure 8E). Thus, short telomeres in human primary cells show evidence of increased telomere recombination, even in the presence of limiting telomerase. This data suggests that some telomere maintenance mechanisms may occur in human cells without a substantial increase in the telomere length distribution.

## Discussion

### Short Telomeres Initiate Recombination in Primary Mammalian Cells

Telomere lengthening is predominantly carried out by telomerase, however other mechanisms including recombination can contribute to telomere length changes. In yeast, short telomeres can stimulate telomere recombination, perhaps due to the loss of telomere capping [24]. Consistent with recombination occurring at short telomeres in mammalian cells, we found an increase in pq-ratio changes and outliers in both telomerase negative and positive cells. Late generation mTR−/− cells with the shortest telomeres showed the greatest amount of pq-ratio changes, and outliers in primary bone marrow and splenocytes. Furthermore, late generation mTR−/− tumor cells were elevated for these changes compared to primary cells. This increase in telomere length fluctuation likely occurs by a recombination-based mechanism, as we found an increased rate of subtelomeric sequence amplification in cells with short telomeres. Thus like in yeast cells, telomeric and subtelomeric recombination is elevated at short, possibly dysfunctional telomeres.

The increase in telomeric recombination occurred even in cells having functional telomerase alleles. Primary cells with short telomeres from CAST/EiJ WT*, mTR+/−, and in human samples with mutations in hTERT showed increased telomere length changes, supporting the idea that telomere recombination mechanisms can occur in the presence of telomerase. Additionally the increase in subtelomeric recombination in late passage mTR+/+ MEFs further demonstrates that both telomerase and recombination can maintain short telomeres. This increased telomere recombination at short telomeres in the presence of telomerase suggest that the telomerase enzyme does not directly contribute to end protection. Thus, the initiation of telomere recombination is more likely due to the disruption of the capping structure at short telomeres, and not the loss of telomerase.

### Multiple Mechanisms Alter Telomere Ratios

Studies of survivors in yeast indicate that telomere recombination can occur by multiple mechanisms. Specifically, two different survivor pathways have been described for telomere recombination. Type I survivors have short telomere tracts and BIR occurs in subtelomeric sequences called Y′ elements. Type II survivors have long telomere tracts and BIR occurs within the telomere repeats themselves [20],[22]. Our findings suggest that mammalian cells also can use various types of recombination mechanisms for telomere maintenance, and that ALT does not occur by a single mechanism. BIR is considered the predominant mechanism in yeast for telomere elongation in survivors [23]. We identified an increased number of metaphases in telomerase deficient lymphomas with subtelomeric recombination, indicative of additional recombination based mechanisms. For instance, degradation of short telomeres into subtelomeric regions likely exposes various types of repetitive sequences. When sequence homology with another chromosome is encountered, strand invasion and copying of the terminal region occurs. The transfer of this unique subtelomeric locus from mouse chromosome 2 to different chromosomes is consistent the possibility of a BIR-like mechanism.

While T-SCEs are typically used as the main measure of telomere recombination in human ALT cells, it is important to note that BIR pathways would not be detected by the CO-FISH method. Although we did detect T-SCE in the bone marrow cells, the number of T-SCEs did not explain the frequency of telomere length variations, indicating others mechanisms must also play a role [33],[34]. In addition, CO-FISH is limited when telomeres are very short, since T-SCEs at short telomeres are very difficult to detect due to the resolution of the telomere probe [35]. In addition, some of the accumulating changes in pq-ratios may also arise as a consequence of stalled replication forks that might be accompanied with dysfunctional telomeres. In *Schizosaccharomyces pombe*, deletion of Taz1, the ortholog of the telomere binding proteins TRF1 and TRF2, can result in replication fork stalling [36],[37]. These studies suggest that dysfunctional telomeres due to the immediate loss of telomere end binding proteins in mammalian cells may result in replication fork stalling, however such intermediates would be predicted to invoke recombination. Thus using pq-ratio and outlier analysis allows detection of various types of recombination.

### Non-Telomerase Mechanisms for Telomere Maintenance in Mouse Cells Have a Minimal Effect on Overall Telomere Length

The use of ALT for telomere maintenance in human cells has characteristically been associated with a dramatic lengthening of telomeres [38]. In contrast, we find in many instances telomere maintenance can occur without extensive telomere elongation. In Eμmyc+mTR−/− G6 transgenic mice, short telomeres dramatically limit tumor growth [7]. However, in a few mice, tumors somehow overcome the short telomeres and continue to grow. When these Eμmyc+mTR−/− tumors were transferred serially through several mice the telomere length was not significantly changed indicating that these tumor cells must utilize non-telomerase mechanisms for telomere length maintenance. However, in sharp contrast to many ALT cell lines, telomeres from these mTR−/− cells did not exhibit a dramatic telomere lengthening. This result of telomere maintenance without significant telomere elongation is very similar to what is seen in Type I survivors in yeast. In Type I survivors the telomeres are very short and yet they are maintained following many doublings and exemplify that not all telomere recombination mechanisms result in dramatic telomere lengthening [21]. In contrast the telomere repeat tracts in Type II survivors are dramatically longer than in wildtype cells. Both Type I and Type II survivors can be generated in a population of cells. Yet due to the growth advantage of Type II survivors in liquid culture, only Type II survivors are seen following extensive growth. The difference between human ALT tumor cell lines and the mouse tumor cells shown here suggests that there may be multiple pathways for recombination in mammals as there are in yeast. One type may predominate in human ALT tumors and immortalized cells and another type may be more favored in primary human cells and mouse cells. Future studies will provide insight into the mechanism of the different pathways and their requirement for the growth of tumors in the absence of telomerase.

## Supporting Information

Figure S1 Q-FISH analysis on various mTR−/− cells. A. Histogram plots of Q-FISH analysis on B-cell lymphomas. B. Histogram plots of Q-FISH on primary bone marrow from myc+mTR+/+ and early and late generation myc+ mTR−/− mice. C. Histogram plots of Q-FISH on primary splenocytes from the same mice as in B. D. Q-FISH on primary bone marrow show that the telomeres of late generation mTR−/−G4 cells are shorter than WT and early generation mTR−/−G1 cells, WT (n = 3), mTR−/−G1 (n = 3), mTR−/−G4 (n = 2). E. Splenocytes from the same mice as in D. This FISH data was used to generate Figures 2 and 3 and Figure S3.
(1.70 MB TIF)

Figure S2 Pq-ratios determined for mouse chromosome 1. A. Shown are the results of a metaphase spread hybridized with mouse chromosome 1 paint probe (green) and a Cy3-labled telomere PNA probe (red). B. The pq-ratios for a myc+mTR+/+ lymphoma are plotted on a log-scale and were typically near 1. C. The pq-ratios for chromosome 1 from a myc+mTR−/−G5 lymphoma are variable with some ratios near 10- and 100-fold.
(5.04 MB TIF)

Figure S3 Pq-ratios on every chromosome for B-cell lymphomas. The pq-ratio plots of every chromosome from multiple metaphases and similar to analysis for mouse chromosome 1 show a dramatic increase in pq-ratio changes in myc+mTR−/−G1 and myc+mTR−/−G6 lymphomas compared to myc+mTR+/+ lymphomas. A and B. Metaphase spreads and pq-ratio plots from myc+mTR+/+ lymphoma hybridized with telomere probe. C and D. Metaphase spreads and pq-ratio plots from myc+mTR−/−G1 lymphoma hybridized with telomere probe. E and F. Metaphase spreads and pq-ratio plots from myc+mTR−/−G6 lymphoma hybridized with telomere probe.
(7.73 MB TIF)

Figure S4 CAST/EiJ mTR−/− splenocytes are increased in pq-ratio changes and outliers. Shown is the telomere length analysis of splenocytes from the mice described in Figure 6. A. Histogram plots of Q-FISH from the progeny of a HG1×HG1 cross: WT2*, HG2 and KO_(G2)_. B. Histogram plots of Q-FISH from progeny of a HG5×HG5 cross WT6*, HG6 and KO_(G6)_. C. Southern analysis on samples from CAST/EiJ splenocytes of the genotypes shown in A and B. D. The percent of chromosomes with a telomere ratio value q/p≥5-fold is plotted on the left y-axis. T-tests (α = .05) on pq-ratio analysis of splenocytes shows a statistically greater amount of telomeres with ratio values of q/p≥5-fold in HG6 cells in comparison to WT (P = .007). T-tests (α = .05) comparing WT cells with mTR−/−_(G2)_ (P = 4×10^−9^) and mTR−/−_(G6)_ (P = 1.4×10^−13^) show a significantly greater amount of q/p ratios≥5-fold for KO_(G2)_ and KO_(G6)_ Black circles represent the mean telomere length plotted on the right y-axis. Error bars represent the SEM. E. The total number of outliers per metaphase is plotted on the left y-axis. A Wilcoxon rank sum test was used to test statistical significance (α = .05) between WT and KO_(G6)_, however no statistical significance was observed (P = 0.9). Black circles represent the mean telomere lengths plotted on the right y-axis. Error bars represent the SEM.
(5.74 MB TIF)
