# Supplementary figures and images for: Short Telomeres Initiate Telomere Recombination in Primary and Tumor Cells
Source: PLoS Genet. 2009 Jan 30;5(1):e1000357. doi: 10.1371/journal.pgen.1000357 (PMC2627939; doi:10.1371/journal.pgen.1000357)

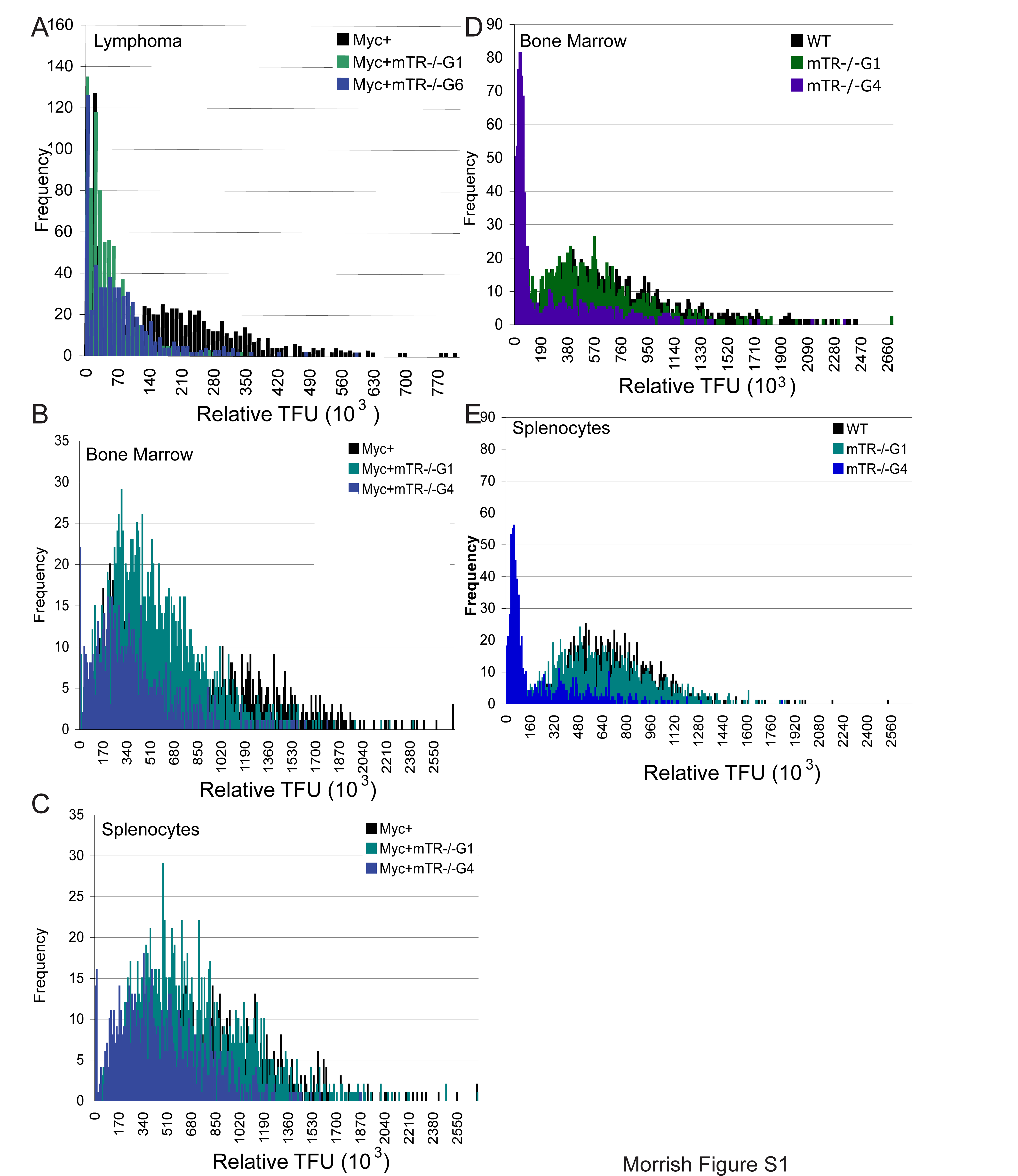

Supplement: Figure S1 — Q-FISH analysis on various mTR−/− cells. A. Histogram plots of Q-FISH analysis on B-cell lymphomas. B. Histogram plots of Q-FISH on primary bone marrow from myc+mTR+/+ and early and late generation myc+ mTR−/− mice. C. Histogram plots of Q-FISH on primary splenocytes from the same mice as in B. D. Q-FISH on primary bone marrow show that the telomeres of late generation mTR−/−G4 cells are shorter than WT and early generation mTR−/−G1 cells, WT (n = 3), mTR−/−G1 (n = 3), mTR−/−G4 (n = 2). E. Splenocytes from the same mice as in D. This FISH data was used to generate Figures 2 and 3 and Figure S3. (1.70 MB TIF) [file pgen.1000357.s001.tif]

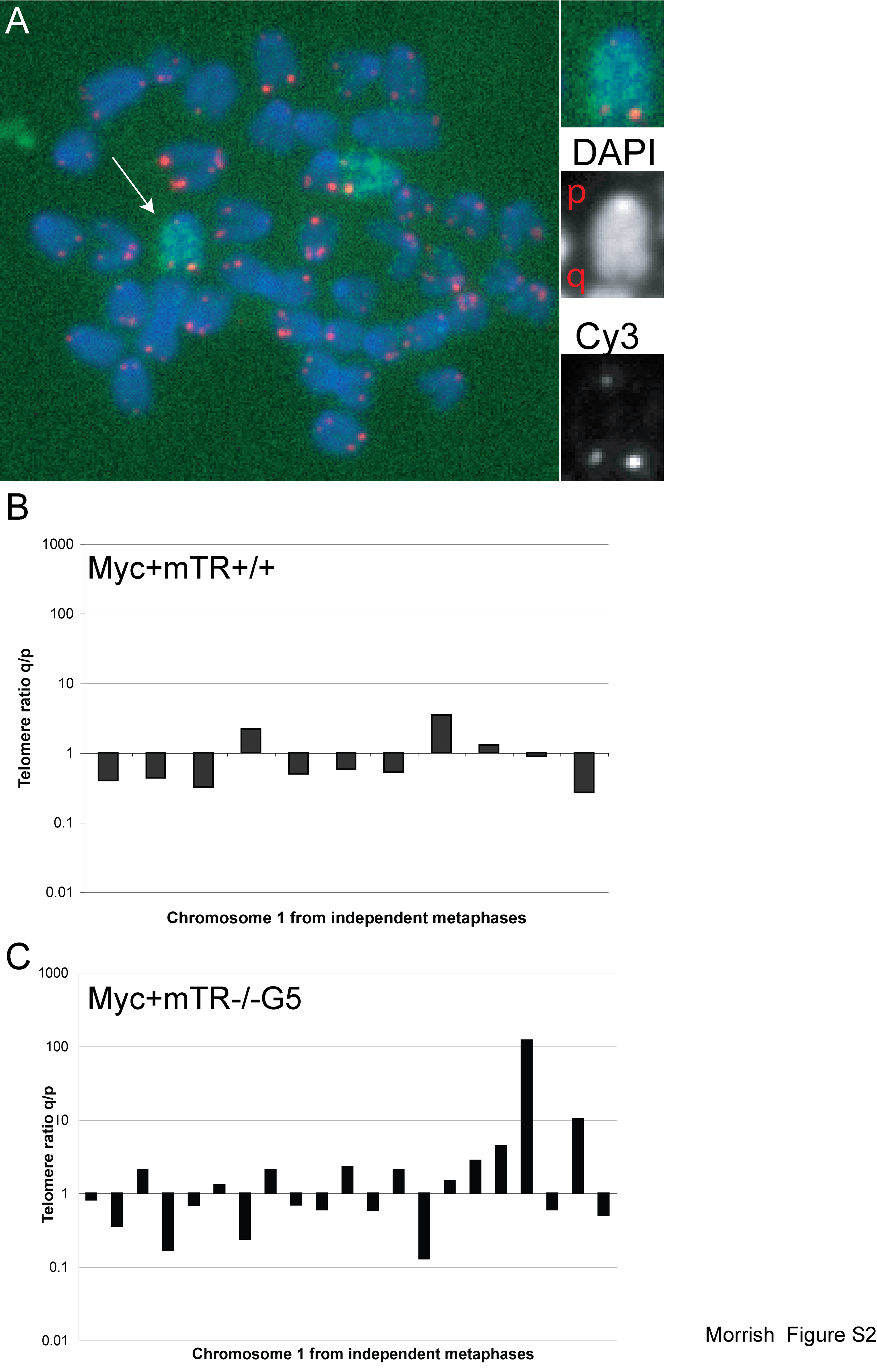

Supplement: Figure S2 — Pq-ratios determined for mouse chromosome 1. A. Shown are the results of a metaphase spread hybridized with mouse chromosome 1 paint probe (green) and a Cy3-labled telomere PNA probe (red). B. The pq-ratios for a myc+mTR+/+ lymphoma are plotted on a log-scale and were typically near 1. C. The pq-ratios for chromosome 1 from a myc+mTR−/−G5 lymphoma are variable with some ratios near 10- and 100-fold. (5.04 MB TIF) [file pgen.1000357.s002.tif]

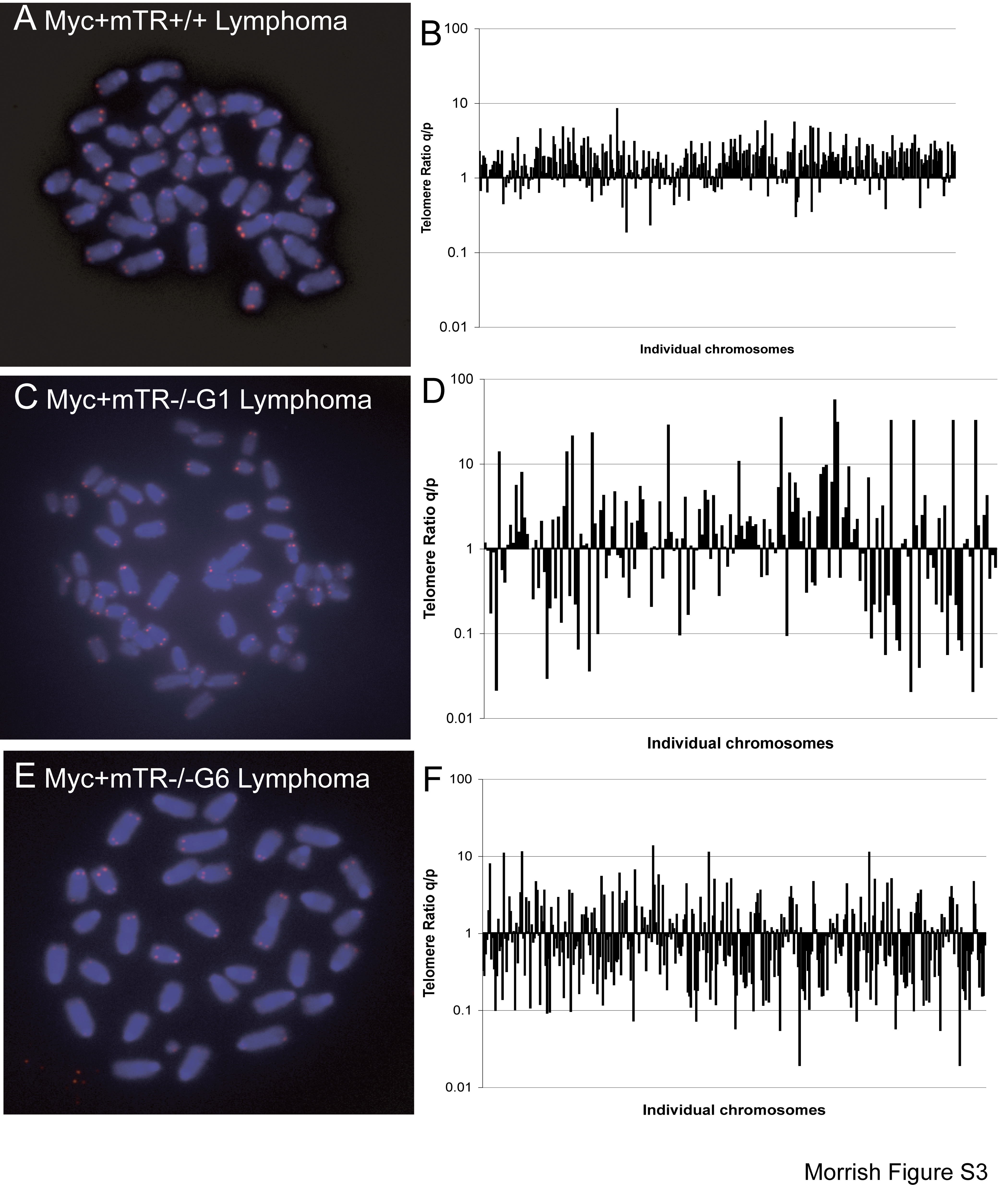

Supplement: Figure S3 — Pq-ratios on every chromosome for B-cell lymphomas. The pq-ratio plots of every chromosome from multiple metaphases and similar to analysis for mouse chromosome 1 show a dramatic increase in pq-ratio changes in myc+mTR−/−G1 and myc+mTR−/−G6 lymphomas compared to myc+mTR+/+ lymphomas. A and B. Metaphase spreads and pq-ratio plots from myc+mTR+/+ lymphoma hybridized with telomere probe. C and D. Metaphase spreads and pq-ratio plots from myc+mTR−/−G1 lymphoma hybridized with telomere probe. E and F. Metaphase spreads and pq-ratio plots from myc+mTR−/−G6 lymphoma hybridized with telomere probe. (7.73 MB TIF) [file pgen.1000357.s003.tif]

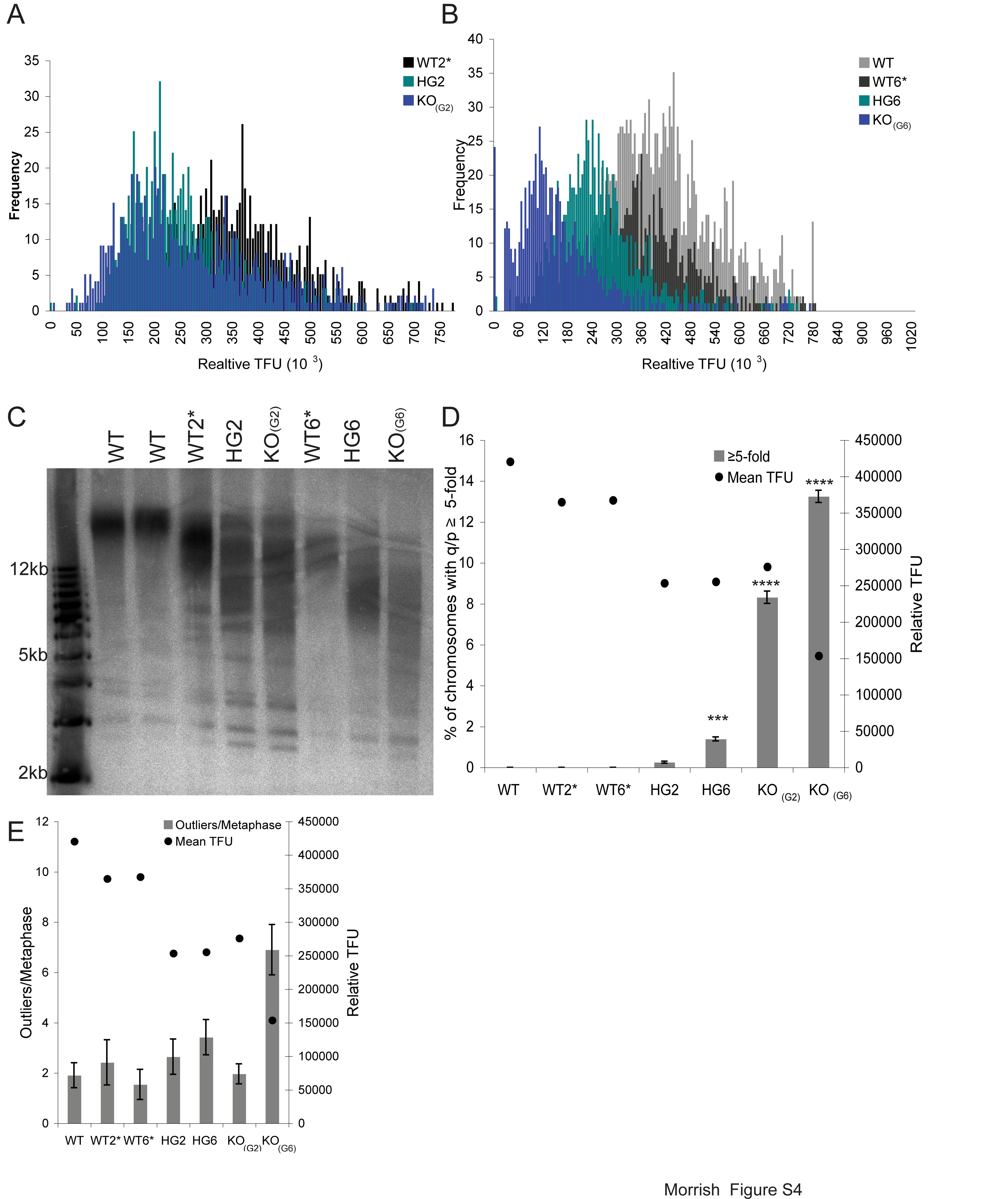

Supplement: Figure S4 — CAST/EiJ mTR−/− splenocytes are increased in pq-ratio changes and outliers. Shown is the telomere length analysis of splenocytes from the mice described in Figure 6. A. Histogram plots of Q-FISH from the progeny of a HG1×HG1 cross: WT2*, HG2 and KO(G2). B. Histogram plots of Q-FISH from progeny of a HG5×HG5 cross WT6*, HG6 and KO(G6). C. Southern analysis on samples from CAST/EiJ splenocytes of the genotypes shown in A and B. D. The percent of chromosomes with a telomere ratio value q/p≥5-fold is plotted on the left y-axis. T-tests (α = .05) on pq-ratio analysis of splenocytes shows a statistically greater amount of telomeres with ratio values of q/p≥5-fold in HG6 cells in comparison to WT (P = .007). T-tests (α = .05) comparing WT cells with mTR−/−(G2) (P = 4×10^−9^) and mTR−/−(G6) (P = 1.4×10^−13^) show a significantly greater amount of q/p ratios≥5-fold for KO(G2) and KO(G6) Black circles represent the mean telomere length plotted on the right y-axis. Error bars represent the SEM. E. The total number of outliers per metaphase is plotted on the left y-axis. A Wilcoxon rank sum test was used to test statistical significance (α = .05) between WT and KO(G6), however no statistical significance was observed (P = 0.9). Black circles represent the mean telomere lengths plotted on the right y-axis. Error bars represent the SEM. (5.74 MB TIF) [file pgen.1000357.s004.tif]
